# Adhesion and running speed of a tropical arboreal ant (*Cephalotes atratus*) on wet substrates

**DOI:** 10.1098/rsos.181540

**Published:** 2018-11-14

**Authors:** Alyssa Y. Stark, Stephen P. Yanoviak

**Affiliations:** 1 Department of Biology, University of Louisville, 139 Life Sciences Building, Louisville, KY 40292, USA; 2 Smithsonian Tropical Research Institute, Balboa, Republic of Panama

**Keywords:** performance, wettability, locomotion, Formicidae, Panama

## Abstract

In the tropical forest canopy, wingless worker ants must cling to and run along diverse vegetative surfaces with little protection from sun, wind and rain. Ants rely in part on their tiny adhesive tarsal pads to maintain sufficient contact with substrates to prevent falls under these varied conditions. Here, we examined the effects of substrate wettability and surface water on the tarsal pad adhesive performance of a common tropical arboreal ant. Ant adhesion was consistently higher on an intermediately wetting substrate (static water contact angle *ca* 90°) when resisting both perpendicular (normal) force and parallel (shear) force. Normal adhesion was maintained on intermediately wetting and hydrophobic substrates following the addition of rain-mimicking water droplets, whereas shear adhesion declined on all substrate types tested after wetting. Ant running speed was slower on wet substrates. On wood substrates, normal and shear adhesion declined with increasing wetness from dry, to misted, to water-soaked. These differences probably contributed to lower ant running speed on wet wood. The results of this study provide the first quantitative assessment of tropical arboreal ant adhesive performance under substrate conditions that are commonly encountered in the rainforest canopy.

## 1. Introduction

Abiotic conditions and associated environmental variation fundamentally determine species distributions. Organisms use diverse behavioural, morphological and physiological mechanisms to deal with such variability [1]. For example, wet surfaces are ubiquitous and often unavoidable in the eponymous rainforest. Whereas many small cursorial organisms have wings that allow them to escape these potentially unsuitable substrates, wingless organisms are constrained to either traverse transiently wet surfaces or to remain immobile until surfaces are sufficiently dry for safe locomotion. For canopy-dwelling taxa, this presents a non-trivial problem, because a 30 m fall is a significant displacement [2–4]. Key among such taxa are ants, which are abundant and ecologically important inhabitants of tropical forest canopies [5].

Arboreal and semi-arboreal ants run along tree branches, vines and leaves when foraging or defending their resources (e.g. [6–8]). These ants depend on the simultaneous functioning of tarsal claws, smooth tarsal pads and a multi-component adhesive secretion [9,10], to prevent falling and thus successfully exploit the canopy environment. By contrast, beetles and other insects use a combination of claws and tarsal fibrils covered with adhesion-mediating fluid to attach [9–12]. Regardless of differences in adhesive mechanism, smooth and fibrillar insect adhesive systems perform similarly [13]. Adhesive insects are ubiquitous and have significant potential as models for bioinspired engineering, yet despite high global insect diversity, they remain relatively unexplored [9]. In particular, ants make up approximately 25% of the terrestrial biomass in the tropics [14] and few studies have quantified how ants function in their environment, including variation in adhesive performance and foraging behaviours under a range of natural environmental conditions (e.g. [7,8,15–17]).

**Figure 1.**
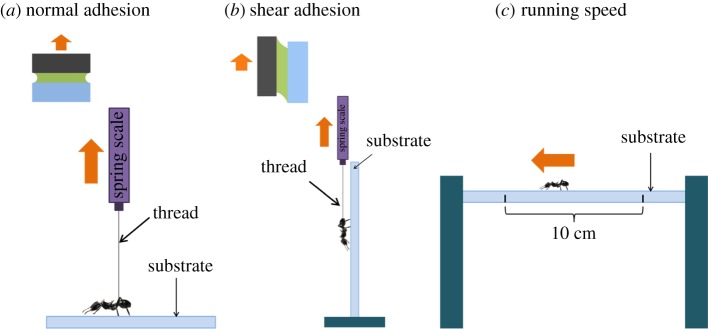
Schematic of the static normal adhesion (*a*), static shear adhesion (*b*) and running speed (*c*) experimental methods. Arrows indicate the direction of pull in (*a*,*b*), and direction of running in (*c*). Insets in (*a*,*b*) depict the contact interface where the fluid-covered tarsi (green = fluid, tarsi = grey) contact the substrate (blue). Arrows indicate loading direction.

Arboreal ants exhibit diverse behaviours that are highly dependent upon effective tarsal adhesion (e.g. manipulation of relatively large loads, subduing prey and avoiding displacement by aggressors or wind). Arboreal ants must resist both perpendicular (normal) and parallel (shear) forces while performing such tasks to avoid being dislodged from canopy substrates (figure 1). During normal adhesion, adhesion-mediating tarsal pad secretions behave similar to a bridge (i.e. capillary-like forces), joining two substrates together (figure 1*a* [10]). Normal adhesion is principally governed by the surface tension and viscosity of the tarsal pad fluid [10,11]. In shear adhesion (figure 1*b*), ants rely on the fluid viscosity of the adhesion-mediating secretion and friction from the tarsal pad to resist high shear loads [11,18,19]. Thus, the term ‘shear adhesion’ includes both an adhesive force from shearing viscous glue (i.e. lubricated sliding [20]) and friction from the tarsal pad. Normal and shear adhesion also contribute to the rapid attachment and release of the tarsal pads during temporary adhesion (i.e. walking and running [12]). In this context, normal and shear adhesion are required to counter the gravitational force of overturning and to apply the appropriate biomechanical loading requirements to engage and disengage the adhesive pad [10,21,22].

**Figure 2.**
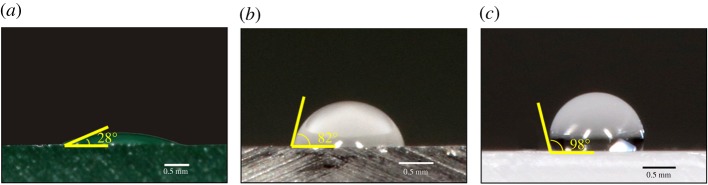
Example static water contact angle (SWCA) droplet images on hydrophilic glass (*a*), intermediately wetting polycarbonate (*b*) and hydrophobic polypropylene (*c*).

In addition to the biomechanical challenges posed by clinging, walking and running, the adhesive system of a tropical arboreal ant must function effectively in diverse environmental conditions. Natural substrates are covered with varying amounts of debris, have variable roughness (e.g. smooth leaves and rough bark) and variable wettability (e.g. hydrophobicity) [6,7,23,24]. Adhesion to these complex substrates probably becomes more difficult during the climatic shifts in temperature, humidity and precipitation that commonly occur in tropical forests. For example, after a rainstorm, substrates that differ in wettability may attract water droplets or water layers to the surface (hydrophilic), or repel water (hydrophobic; figure 2).

The principal goal of this study was to determine the effect of substrate wettability and the presence or absence of water on ant adhesive performance. We hypothesized that the adhesive performance of tropical arboreal ant tarsal pads is affected by substrate wettability under both wet and dry surface conditions, in a manner similar to other biological and synthetic adhesive systems [25–29]. We also measured adhesive performance on natural wood substrates ranging in wetness from dry to saturated to replicate the conditions ants encounter in the forest. Finally, because ants use their adhesive system for multiple functions, we separated adhesive performance into three forms: static normal adhesion (i.e. vertical detachment), static shear adhesion (i.e. sliding) and temporary adhesion measured as running speed (hereafter referred to as running speed; figure 1). We explored this problem with a series of field and laboratory experiments focusing on a common tropical arboreal ant species.

## 2. Material and methods

Field and laboratory experiments were conducted on Barro Colorado Island (BCI), Panama (9.15° N, 79.85° W) during the 2015 wet season (May–July). More information about this site is available elsewhere [30,31]. We used workers of the tropical ant species *Cephalotes atratus* L. (Hymenoptera, Formicidae, Myrmicinae) for all experiments. We chose this species because it is common on BCI, is readily recognized, has large workers (*ca* 43 mg) that are easily handled and has an arboreal lifestyle. Like other arboreal ants, *C. atratus* workers adhere using tarsal pads (tarsal pad area *ca* 0.0140 mm^2^) and tarsal claws, which can grip rough substrates.

### 2.1. Experimental substrates

We measured static adhesion and running speed of *C. atratus* workers on three substrates that probably approximate the variety of substrate wettability ants experience in their natural habitat (i.e. smooth glass (Kentucky Mirror & Plate Glass, Louisville, KY), polycarbonate (PC; U.S. Plastic Corporation, Lima, Ohio) and polypropylene (PP; U.S. Plastic Corporation, Lima, Ohio)). We also measured adhesion on commercially available kiln-dried red oak (*Quercus* spp.) to represent a natural substrate. Static adhesion was measured on flat substrates, whereas running speed was measured on 1 cm diameter dowels of the same material and manufacturer as the flat substrates. We found ants were more likely to run on the dowels than the flat substrates, and their running direction was limited to a linear trajectory when running on dowels. Ant adhesion and running speed was measured on each substrate first under dry conditions and then after applying a fine, uniform spray of water droplets from a water bottle (hereafter, wet conditions).

To quantify substrate wettability, we measured the static water contact angle (SWCA [29]) of distilled water (*ca* 0.01 ml applied via a 1 ml syringe) on each of the flat substrates (figure 2). Contact angles were measured from digital photographs of water droplets with NIH ImageJ version 1.46r software. SWCA was not measured on the wood surfaces because wood fibres absorb water, making contact angle measurements impossible. SWCA was measured by averaging the right and left angles the water droplet makes with the substrate surface (figure 2). Two images per droplet were used, and droplets were measured at three different locations on each substrate. Thus, the average SWCA of each substrate was calculated from 12 measurements (i.e. right and left sides, two images per location, three locations per substrate).

Preliminary field observations during and after rain events (*ca* 30 min post rain) showed that ants are rarely active during rain events, but will forage after rain; however, their ability to traverse rain-soaked wood appeared to be diminished (electronic supplementary material, movie S1) relative to locomoting on dry wood, and wood with water droplets distributed across its surface. To investigate static adhesion and running speed to water-saturated wood, we soaked wood substrates in water for more than 3 h and immediately measured ant adhesion on the wood upon removal from the water. These experiments were conducted over 60 min, and the wood substrate was re-submerged periodically (*ca* every other individual) to ensure the wood remained saturated with water across all experimental trials. Adhesion to soaked wood did not change as a function of time in shear or normal adhesion tests (*F*_1,13_ = 0.8174, *R*^2^ = 0.06, *p* = 0.3824; *F*_1,13_ = 0.1562, *R*^2^ = 0.01, *p* = 0.6991, respectively).

### 2.2. Adhesion experiments

*Cephalotes atratus* workers used in experiments were collected by hand from at least two colonies during normal foraging activities. We used only individuals that were intact, able to articulate all legs and able to cling to or run across the experimental substrates. Ants were allowed to acclimate to laboratory conditions for at least 1 h (*ca* 22°C and 79% RH) before measurements were taken. Each ant was weighed to the nearest 0.1 mg and returned to the colony at the end of the experiment.

Static adhesion was quantified by looping a thin nylon thread (Aurlfil; Milano, Italy) around the petiole of the ant and attaching the thread to a 10 g capacity spring gauge (Pesola; Schindellegi, Switzerland). The flat test substrate was oriented either horizontally or vertically for normal and shear adhesion tests, respectively (figure 1 [16]). Prior to adhesion testing, ants were allowed to attach naturally by taking a few steps on the substrate. Each individual (*N* = 15) was tested in the normal and shear loading orientation three times for each substrate type and water treatment, totalling six static adhesion measurements per ant. Only the highest normal and shear adhesion values from each trial were used in analyses. Adhesion was defined as the maximum load (in grams) that an ant resisted when: (i) being pulled perpendicular to the substrate in normal adhesion tests (to the point of detachment), or (ii) sliding along the substrate for *ca* 3 cm in shear adhesion tests. In all adhesion experiments, ants were pulled by hand by the same experimenter for consistency. Surfaces were cleaned with ethanol, then water and subsequently towel-dried after each series of trials for a given ant [16].

Running speed was measured on 1 cm diameter rods elevated using a simple wood frame (figure 1*c*). Speed was measured as the maximum running speed (cm s^−1^) across a 10 cm section of the rod substrate. Running speed was timed using a hand-held stopwatch, and only complete, uninterrupted, straight runs on the top surface of the dowel were used [7]. Each ant (*N* = 30) was tested consecutively three times on each substrate and treatment condition. The maximum speed recorded from three consecutive 10 cm runs for a given ant was used in statistical analysis.

### 2.3. Statistical analysis

Static normal adhesion, static shear adhesion and running speed were analysed separately. We used an analysis of covariance (ANCOVA) to test for differences in static adhesion and running speed versus treatment (wet and dry) on glass, PC and PP. Similarly, we compared static adhesion and running speed on the wood substrate when wet, dry and soaked. Ant mass was used as the covariate. We used a Tukey HSD test to assess differences among means when ANCOVA results were significant. Data were log transformed to meet statistical assumptions. Analyses were conducted using JMP 10 software (SAS Institute, Inc. 2012) and means are reported ± s.e.

## 3. Results

### 3.1. Substrate wettability

SWCA measurements revealed that the three smooth artificial substrates varied in substrate wettability. The SWCA of hydrophilic glass was 27.3 ± 3.39° (i.e. SWCA < 90°). The SWCA of PC was 85.5 ± 1.59°, which is within the expected range (*ca* 90°). Finally, the SWCA of PP was 99.7 ± 0.46°, which is operationally defined as hydrophobic (i.e. SWCA ≥ 90°). The static water contact angles measured in this project were similar to values reported elsewhere for the same substrates [32].

### 3.2. Adhesion to smooth artificial substrates

**Figure 3.**
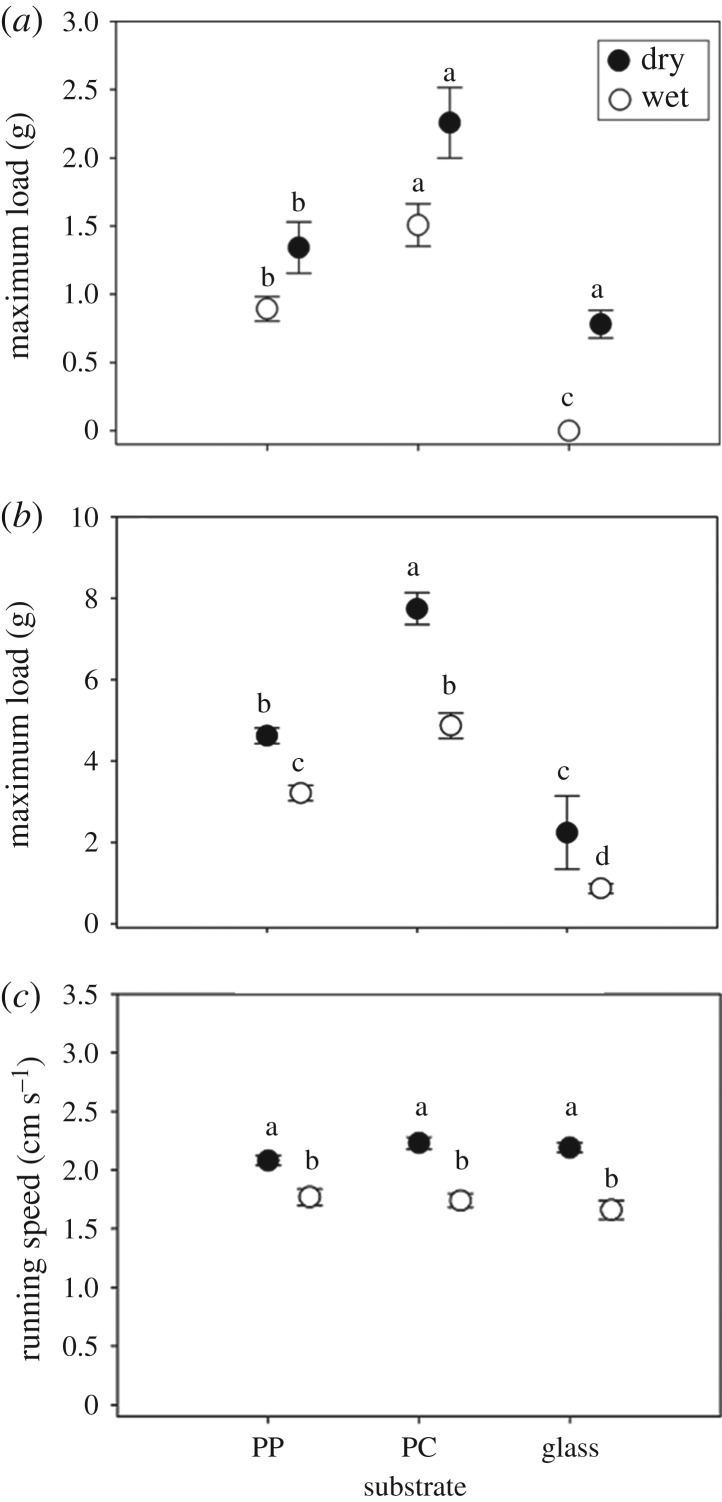
Static normal adhesion (*a*), static shear adhesion (*b*) and running speed (*c*) on three smooth, artificial substrates that vary in substrate wettability (static water contact angle *ca* 27°, 85° and 100° for glass, polycarbonate (PC) and polypropylene (PP), respectively). Treatment groups denoted with the same letter are not significantly different.

Normal and shear adhesion of *C. atratus* on smooth artificial substrates (i.e. glass, PC and PP) differed in response to substrate wettability and the presence or absence of water (normal adhesion: *F*_11,78_ = 16.40, *p* < 0.0001; shear adhesion: *F*_11,78_ = 49.25, *p* < 0.0001). Specifically, normal adhesion in the air was higher on PC (SWCA = 85.5°) than PP (SWCA = 99.7°) and glass (SWCA = 27.3°; figure 3). The addition of water from a spray bottle did not affect adhesion on PC and PP, but adhesion was lower on misted glass than on dry glass (figure 3*a*). Shear adhesion was higher on PC than PP and glass, and was lower on all three substrates when wet (figure 3*b*). Running speed of *C. atratus* on dowels also differed in response to the water treatment (*F*_11,168_ = 8.50, *p* < 0.0001). Specifically, while running speed was unaffected by substrate wettability in air, speed was lower on all substrates after they were misted with water (figure 3*c*).

### 3.3. Adhesion to wood

**Figure 4.**
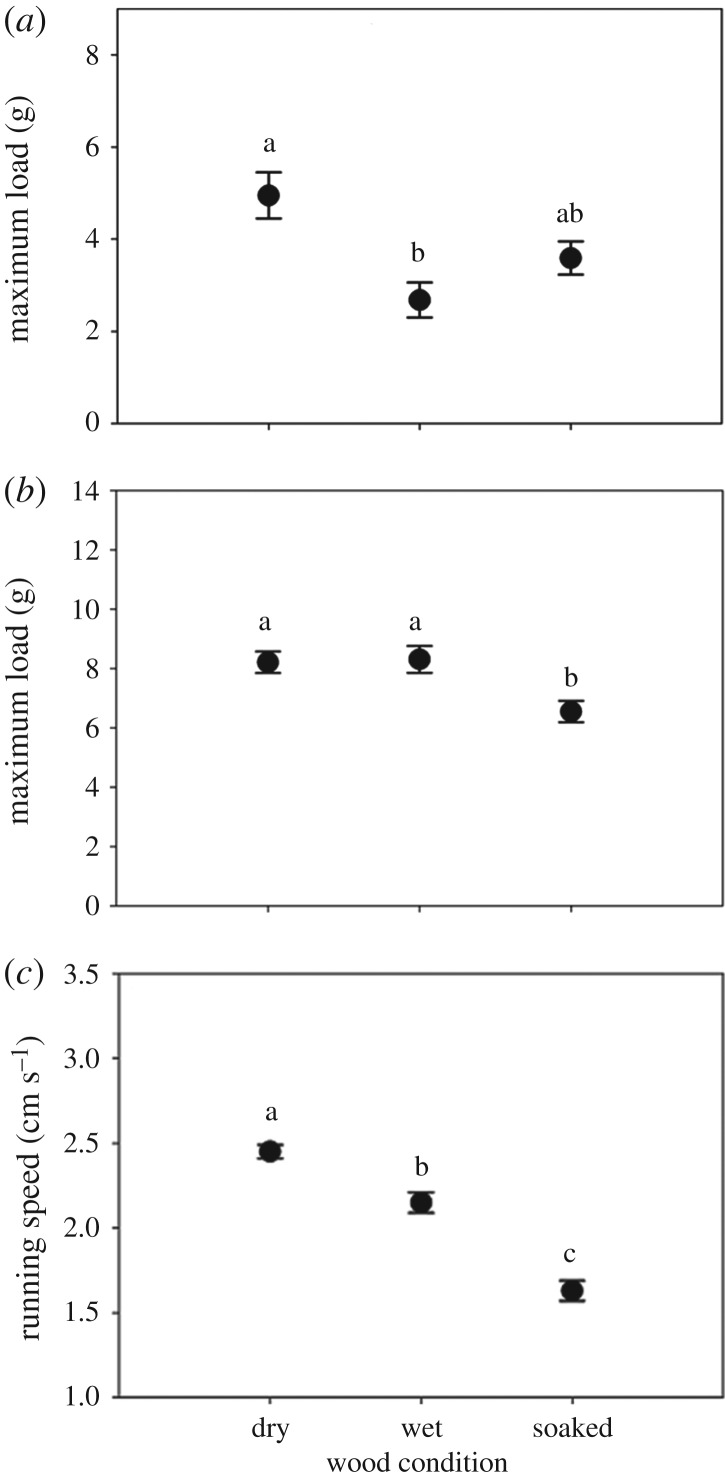
Static normal adhesion (*a*), static shear adhesion (*b*) and running speed (*c*) on dry, misted with water drops (i.e. wet) and water-soaked wood. Treatment groups denoted with the same letter are not significantly different.

Normal and shear adhesion of *C. atratus* on the natural wood substrate differed in response to the water treatment (normal adhesion: *F*_5,39_ = 4.56, *p* = 0.0023; shear adhesion: *F*_5,39_ = 4.24, *p* = 0.0036). Normal adhesion of *C. atratus* was highest on dry wood and lowest on wood misted with water; however, on soaked wood, ants produced forces similar to those on dry or misted smooth synthetic surfaces (figure 4*a*). Shear adhesion of *C. atratus* did not differ between dry and misted wood but was lower on soaked wood (figure 4*b*). Running speed of *C. atratus* also differed among water treatments (*F*_5,84_ = 23.25, *p* < 0.0001); specifically, running speed was fastest on dry wood and slowest on water-soaked wood (figure 4*c*).

## 4. Discussion

Secure attachment to substrates in the tropical forest canopy is important for the survival of arboreal organisms, especially wingless animals. Here we show that workers of a common species of tropical arboreal ant exhibit variation in static adhesion on different experimental substrates and under wet and dry conditions of the substrate. We also found that an ant's ability to maintain speed while running depends on the presence or absence of water on the surface, particularly on wood.

Variability in substrate wettability is common in the tropical forest canopy. For example, leaves, bark and vines can range in wettability from superhydrophobic to hydrophilic [24]. Moreover, most surfaces in the tropical rainforest are covered by lichens, which also exhibit high variation in wettability [33]. Whereas some insects (e.g. specialized herbivores) have adhesive systems tuned to match the wettability of host plant surfaces [34], substrate roughness often overshadows the effect of substrate wettability on insect adhesion to dry surfaces [35–37]. On manufactured surfaces, we found that surface wettability affects ant adhesion in both dry and wet conditions, suggesting that substrate wettability drives ant adhesive performance on relatively smooth surfaces, especially in areas of high humidity and rain. Further work is needed to clarify the effect of substrate wettability on ant adhesion on rough natural surfaces that vary in wettability, like the lichen-covered bark of trees and lianas. Although our study used glass, plastic substrates and refined wood that does not match the surface chemistry or texture of bark, our results suggest that the highly variable conditions and substrate characteristics of the rainforest canopy pose substantial challenges to arboreal ant adhesion, requiring behavioural, physical and chemical mechanisms to circumvent these challenges.

The results of this study complement similar findings in other biological and synthetic systems [25–29] and are probably driven by the interaction of the tarsal pad secretion chemistry and surface energy of the substrate. For example, the presence of specific residue in the ‘footprints’ left by the tarsal pads of Asian weaver ants (*Oecophylla smaragdina* Fab., Hymenoptera, Formicidae, Formicinae) depends on the nature of the surface (i.e. hydrophilic versus hydrophobic glass surfaces) [10]. This suggests some level of ‘tuning’ between the ant tarsal pad secretion and the substrate (e.g. hydrophobic fluid dominates on hydrophobic substrates). Moreover, the combination of hydrophobic and hydrophilic residues in these footprints indicate that ants walking on intermediately wetting substrates like PC can employ an adhesive emulsion of hydrophobic and hydrophilic components to match the intermediate surface energy. It is possible that this combination of fluids allows for improved adhesion on PC when compared to glass and PP. Additional chemical changes probably occur in the presence of water; in the gecko adhesive system water causes a restructuring of chemical groups at the adhesive interface [38]. Regardless, more research is needed to clarify the interaction between the chemistry of the tarsal pad fluid, substrate surface energy and presence or absence of water. Although circumstantial, the lack of difference in ant running speed on dry artificial substrates differing in wettability suggests that the ant adhesive system is labile on diverse surfaces, similar to other fast-moving adhesive organisms (e.g. gecko running speed is the same on dry glass and dry acrylic [39,40]).

We believe that ants interact with water in a different way to beetles, geckos, tree frogs, newts and some bioinspired synthetic adhesives who have air pocket-forming fibrils or channels. In these systems, air is either trapped to form a bubble that presses water out of the interface (e.g. beetles, geckos, bioinspired synthetics [25,28,41–45]), or microchannels are used to shunt water out of the interface (e.g. frogs and newts [46–48]). Both mechanisms remove water from the contact interface and allow for dry or near-dry contact. The results of this study clearly indicate that ants, and probably other insects with smooth tarsal pads, are unable to do this, as there are only fine wrinkles on the tarsal pads (see [49] and [50] for images of cockroach and stick insect smooth pads, respectively). Physical limitations of the smooth pad are probably heightened on flooded surfaces, similar to the fibrillar pads of beetles and spiders [51–53], where adhesion-enhancing capillary effects are eliminated due to thick water layers that build up in high humidity, or when ant tarsi encounter standing water from rain. Resolving the physical mechanisms for these patterns was beyond the scope of this study but is a potentially fruitful direction for further research.

Ants rarely encounter smooth, firm substrates (like glass) in nature, thus ant tarsal adhesion is probably a product of forces generated by both the pad and claws. The results of this study suggest that tarsal claws play an important role in attachment to natural substrates like wood (normal adhesion was 2× higher on dry wood versus dry smooth substrates). However, when running, speed decreased significantly when wood became more waterlogged, probably impeding the functionality of the claws and tarsal pads (e.g. reduction of contact area, instability of substrate) [21]. Specifically, changes in surface roughness and mechanical properties of misted and soaked wood may cause claws and pads to lose purchase or to fail. Furthermore, although the time ants were in contact with water-soaked surfaces was short (i.e. the duration of an experiment was *ca* 5 min), it is possible the tarsal pad cuticular material also changed, similar to the water-dependency of gecko and beetle setae [54,55]. The results of this study indicate that worker ants traversing wet canopy substrates are slowed and have a lower adhesive force available to support their body weight in moist conditions. This is important given that the force of rain droplets may be sufficient to dislodge an ant seeking shelter [21,56], and this danger would be heightened when navigating wet surfaces (also see [17]).

Although the results of this study show that the substrate condition has a significant effect on adhesive performance, several questions remain unanswered. For example, what is the range of natural substrate wettability in the tropical forest canopy?; what are the physico-chemical interactions between the substrate, ant claws and adhesive pad?; and what type (shear adhesion, normal adhesion, mechanical interlocking) and magnitude of attachment is needed to resist gravitational overturning with respect to the orientation of the ant (i.e. horizontal, vertical or inverted), biomechanical loading (e.g. walking, running, pulling) and behaviour (e.g. activity during or after rain)? Future investigation into how, when and where ant adhesion is used in the tropical canopy will clarify the significance of environmental challenges such as rain, wind and temperature on ant behaviour and ecology, and improve design parameters for ant-inspired synthetic adhesives.

## Supplementary Material

Movie S1

## Supplementary Material

Data Set 1

## Fieldwork permission

The Smithsonian Tropical Research Institute provided access and permission to conduct field research on Barro Colorado Island.

## Data accessibility

The datasets supporting this article have been uploaded as part of the electronic supplementary material.
